# Objective Measurement of Musculoskeletal Pain: A Comprehensive Review

**DOI:** 10.3390/diagnostics15131581

**Published:** 2025-06-22

**Authors:** Nahum Rosenberg

**Affiliations:** Specialists Center, National Insurance Institute, Pal Yam 8, Haifa 3309511, Israel; nahumrosenebrg@hotmail.com; Tel.: +972-54-4685130; Fax: +972-4-8590930

**Keywords:** musculoskeletal pain, objective assessment, biomarkers, heart rate variability, facial expression, neuroimaging, functional MRI, pain diagnostics, multimodal evaluation

## Abstract

**Background:** Musculoskeletal (MSK) pain is a leading contributor to global disability and healthcare burdens. While self-reported pain scales remain the clinical standard, they are limited by subjectivity and inter-individual variability. Therefore, objective assessment tools are increasingly sought to enhance diagnostic precision, guide treatment, and enable reproducible research outcomes. **Methods:** This comprehensive narrative review synthesizes evidence from physiological, behavioral, and neuroimaging approaches used to evaluate MSK pain objectively. Emphasis is placed on autonomic biomarkers (e.g., heart rate variability, skin conductance), facial expression analysis, electromyographic methods, and functional neuroimaging modalities such as fMRI and PET. Emerging applications of artificial intelligence and multimodal diagnostic strategies are also discussed. **Results:** Physiological signals provide quantifiable correlations of pain-related autonomic activity but are influenced by psychological and contextual factors. Behavioral analyses, including facial action coding systems and reflex testing, offer complementary, though complex, indicators. Neuroimaging techniques have identified pain-related brain patterns, yet clinical translation is limited by variability and standardization issues. Integrative approaches show promise for improving diagnostic validity. **Conclusions:** Objective assessment of MSK pain remains methodologically challenging but holds substantial potential for enhancing clinical diagnostics and personalized management. Future research should focus on multimodal integration, standardization, and translational feasibility to bridge the gap between experimental tools and clinical practice.

## 1. Introduction

Musculoskeletal (MSK) pain, involving various conditions affecting joints, muscles, bones, and connective tissues, presents a significant global health burden. Its impact extends beyond physical discomfort, often leading to functional limitations, reduced quality of life, and substantial socioeconomic costs. Accurate assessment of MSK pain is vital for effective clinical management, guiding treatment decisions, monitoring therapeutic efficacy, and facilitating research to develop novel pain management strategies [1,2]. However, pain’s subjective and multifactorial nature presents considerable challenges to its objective measurement. Unlike other medical conditions with readily quantifiable biomarkers, pain is an inherently personal experience influenced by a complex interplay of biological, psychological, and social factors [3]. Traditional pain assessment relies heavily on patient self-report, using tools like the Numeric Pain Rating Scale, Verbal Rating Scales, and Visual Analog Scales. While valuable, these subjective measures are susceptible to individual biases, cognitive factors, and contextual influences [3]. This inherent subjectivity underscores the pressing need for objective pain assessment tools that can complement patient-reported outcomes and provide a more comprehensive understanding of the pain experience [4,5]. Furthermore, the multidimensional nature of chronic MSK pain requires a multi-branched assessment approach to capture its diverse aspects [6].

This review explores the current knowledge of objective MSK pain measurement techniques, examining various physiological, behavioral, and neuroimaging approaches and the underlying principles of each method, discussing their strengths, limitations, and potential applications in clinical practice and research. The challenges and future directions in the ongoing search for more clinically relevant objective pain assessment tools will be discussed. The development and standardization of such tools hold immense promise for improving the lives of patients with MSK pain [7,8]. This includes aiding diagnosis and personalized treatment strategies and facilitating more precise and comparable outcome measures in clinical trials, thereby accelerating the development of more effective pain management interventions [5].

## 2. Physiological Approaches to Pain Measurement

One prominent strategy in objective pain assessment involves monitoring the physiological markers associated with the pain. These physiological markers reflect the activation of the autonomic nervous system, which is modulated by pain perception [9]. However, it is essential to remember that while physiological changes can accompany pain, they do not directly measure the subjective experience of pain [10]. Furthermore, patient self-reporting remains a cornerstone of pain assessment, even alongside physiological measures [11]. Therefore, the limitations of current objective measures emphasize the need for ongoing research and development [12]. One approach measures change in the autonomic nervous system, such as heart rate variability, skin conductance, and pupillary responses. These parameters have been shown to correlate with pain intensity and can be monitored non-invasively [13]. For example, studies have reported that heart rate variability (HRV) can be a pain marker under general anesthesia [14]. However, relying solely on physiological signals should be done cautiously, and additional measures should be advocated [13]. Furthermore, factors such as medication, underlying conditions, and psychological states can influence these measures, complicating their interpretation.

Another physiological manifestation of pain is altered peripheral blood flow, which can be measured using laser Doppler flowmetry and thermal imaging techniques. These methods can detect changes in skin blood flow in response to painful stimuli, reflecting underlying physiological mechanisms of pain. Changes in peripheral blood flow are a recognized physiological response to pain, potentially offering a non-invasive assessment method. This can be achieved by utilizing laser speckle flowgraphy to evaluate blood flow changes, in the context of pain’s local or general management [15,16]. An additional method for pain’s quantification is assessing microcirculation, which is applied in studies of fibromyalgia patients who experience chronic pain [16].

It has been suggested that the perfusion index (PI), derived from pulse oximetry, be used to indicate vasomotor disturbances, which are often associated with pain conditions, such as complex regional pain syndrome (CRPS). While not directly measuring blood flow, PI provides information about peripheral perfusion related to blood flow [17].While PI is not exclusively a pain marker and primarily reflects vasomotor activity, research suggests that it may have potential as an adjunctive tool in pain assessment, particularly in specific contexts such as postoperative pain or CRPS. However, further research is needed to validate its reliability and specificity for pain assessment across diverse populations and pain conditions

Thermal imaging is used in pain assessment, as it can detect changes in skin temperature related to inflammation and altered blood flow. These techniques can provide valuable insights into the physiological changes associated with pain, but they are not always specific to pain itself and can be influenced by other factors. Interpreting these measurements requires careful consideration of the clinical context.

### 2.1. Autonomic Nervous System Responses

Several studies have investigated using autonomic nervous system responses as markers of musculoskeletal pain [12]. These measures include changes in heart rate, heart rate variability, blood pressure, and skin conductance. The underlying evidence is that pain stimuli activate the sympathetic nervous system, leading to measurable physiological changes. It was found that pain stimuli increase the heart rate, suggesting its potential utility in assessing pain [18]. However, cardiovascular responses, such as heart rate and blood pressure, can be heterogeneous and may not consistently demonstrate consistent reactions to pain. More precise measurement techniques, such as those assessing peripheral vasoconstriction and sympathetically driven cardiac responses, e.g., HRV, have revealed more consistent increases in sympathetic activity during pain [19].

Skin conductance, an electrodermal activity that reflects sympathetic nervous system activation, has consistently increased during painful stimulation [20]. This method responds quickly to pain stimulus and is independent of changes in hemodynamics, temperature, breathing, or neuromuscular blockade. Its sensitivity reaches 90%, with a specificity up to 74% [21].

While these physiological changes can be informative, it is essential to consider the influence of various factors on autonomic nervous system (ANS) responses. Psychological factors such as imagery, social impact, hypnosis, attention, predictability, and anxiety can modulate the autonomic component of pain’s expression. Even the perceived unpleasantness of a stimulus can influence autonomic responses. Thus, ANS responses offer potential as objective pain markers and should be interpreted cautiously, considering the multifaceted nature of pain and the possible influence of confounding factors, including psychological ones [19,22].

While both ECG and pulse oximetry provide valuable physiological data, their use for objective musculoskeletal pain assessment is not currently supported by robust clinical evidence. ECG can detect changes in heart rate and variability, which may be influenced by pain, but these changes are neither specific to pain nor its musculoskeletal origin [19]. Similarly, pulse oximetry measures blood oxygen saturation, a vital sign that can be affected by various factors unrelated to pain [23]. Although physiological responses like heart rate or respiration changes can occur during pain, they are not reliable or specific enough for objective pain quantification, especially for musculoskeletal issues.

Research exploring multi-parametric approaches incorporating various physiological signals, including electrodermal activity, holds some promise [24], but further investigation is needed to determine their clinical utility for musculoskeletal pain assessment. Therefore, relying solely on ECG and pulse oximetry for objective musculoskeletal pain measurement is not currently recommended, due to its complex and multifactorial nature beyond these physiological markers [2].

### 2.2. Muscle Activity and Nociceptive Flexion Reflexes

Changes in muscle activity and nociceptive flexion reflexes have also been explored as potential objective markers of musculoskeletal pain. The nociceptive flexion reflex (NFR), a withdrawal reflex evoked by noxious stimuli, has been studied as a measure of spinal nociceptive processing as a valuable tool for studying the impact of various interventions on pain processing, both in acute pain and healthy individuals [25]. This reflex, also known as the R-III reflex, involves the activation of nociceptive afferents, leading to a flexion withdrawal response [26]. An additional electrophysiologic diagnostic tool, utilizing electromyographic (EMG) recordings of muscle activity, has been suggested to assess pain-related changes in animal studies [27]. EMG can measure muscle tension during painful stimulation, showing consistent increases in muscle activity with more intense and uniform stimuli [27]. EMG’s physiological modulation of nociceptive transmission and processing might be monitored when pain is associated with a neurological deficit [28]. There is evidence that in patients with symptomatic lumbar spinal stenosis, the paraspinal mapping EMG score had a 100% specificity and 30% sensitivity diagnostic rates [29]. However, electrophysiologic methods are limited because of differences in individual assessments [30].

Therefore, while these physiological measures provide valuable insights into the neurophysiological mechanisms underlying musculoskeletal pain, their clinical applicability is limited. Further research is needed to standardize these methods and establish normative data, enabling reliable individual assessments in clinical practice. International efforts aim to standardize outcome measures in chronic pain trials. The Initiative on Methods, Measurement, and Pain Assessment in Clinical Trials (IMMPACT) is a significant international effort dedicated to improving the design, execution, and interpretation of clinical trials for chronic pain treatments [31]. A primary goal of IMMPACT is to establish core outcome domains that should be consistently assessed in chronic pain clinical trials [32]. These domains include pain intensity, physical functioning, emotional functioning, participant ratings of improvement and satisfaction with treatment, symptoms and adverse events, and reasons for withdrawal from the study.

Currently, IMMPACT is used as a standard reference point for designing pain research and interpreting the results of clinical trials.

## 3. Behavioral Measures of Musculoskeletal Pain

In clinical practice, pain not only affects function but also mediates and distorts core outcome metrics such as joint mobility, muscle strength, and movement quality. As such, these metrics, commonly used to track recovery, can simultaneously be seen as artifacts altered by pain and as indirect indicators of pain’s presence or modulation [33]. A valid and reliable assessment of pain is fundamental for both clinical trials and effective pain management [5]. Despite ongoing efforts, achieving an objective measure of pain is challenging due to its complex and subjective nature, and a single, definitive method has yet to be established. [5].

Behavioral measures, especially involving facial expressions, potentially offer a valuable approach to objectively assessing musculoskeletal pain. These methods provide observable indicators of pain that can complement self-report measures and offer insights into the impact of pain on functional activities [34].

Facial expressions are intrinsically linked to the subjective experience of pain and have been extensively studied as potential objective markers. The ability to recognize and interpret facial expressions related to pain has implications for understanding how pain is perceived and judged by observers [34].

Paul Ekman and Wallace Friesen developed the Facial Action Coding System (FACS) to taxonomize human facial expressions [35]. It is a comprehensive, anatomically based system that describes all visually discernible facial movements. FACS is not explicitly designed for pain; it can be used to describe any facial expression. FACS breaks down facial expressions into components called Action Units (AUs). Each AU corresponds to the contraction of specific facial muscles. For example, AU 4 corresponds to brow lowering, AU 6 to cheek raising, and AU 7 to eyelid tightening. These AUs can occur in isolation or in combination to create a wide range of expressions. While FACS itself is a neutral system for describing any facial movement, researchers have used it to identify AUs consistently associated with pain. It has been suggested that different AUs are more frequent and intense during painful experiences [36,37]. These AUs are not exclusively indicative of pain but can also occur in other emotional states, like sadness or fear. However, their presence, particularly in specific combinations and intensities, provides valuable clues for assessing pain, especially when verbal communication is limited [37].

One of FACS’ strengths is its objectivity and detailed descriptions of facial muscle activity. This helps standardize facial expression observations, reducing subjectivity and making comparisons across studies more reliable. However, FACS is complex and requires extensive training to use reliably. Manual coding is also time-consuming. The automated facial expression analysis systems promise to make this process more efficient, although these systems are still under development [38]. Currently, it is difficult to provide specific sensitivity and specificity values for facial expression analysis (particularly using the Facial Action Coding System—FACS) in pain evaluation, because FACS is primarily a descriptive tool that identifies and quantifies facial muscle movements. Its utility in pain assessment depends on how these AUs are interpreted and linked to pain [37].

Advancements in computer vision and machine learning have facilitated the development of automated facial expression analysis systems. These systems can automatically classify facial expressions associated with pain, potentially enabling real-time monitoring and assessment. The use of multimedia processing for automatic pain assessment from facial and bodily expressions highlights the growing role of technology in this field [39].

Several studies have explored the use of wearable devices for facial expression analysis [40,41]. For example, glasses-type wearable devices with cameras can capture facial expressions [42]. Additionally, facial EMG sensors can monitor muscle activity related to different emotions [40]. An ear-worn device has also been proposed for facial emotion recognition disorders [41]. Overall, the integration of facial expression analysis with wearable or real-time monitoring systems has the potential to revolutionize clinical practice by providing objective, continuous, and personalized insights into patients’ emotional and physical states [43]. While not yet standard practice, facial expression analysis using wearable systems is gaining traction in clinical research and shows potential for various applications, particularly in remote monitoring and personalized treatment [41,44]. Further research and development are necessary to overcome existing challenges and facilitate the widespread clinical adoption of this technology.

While facial expression analysis offers numerous advantages, such as being non-invasive and potentially enabling real-time monitoring, it also has limitations. Facial expressions can be influenced by individual, cultural, and cognitive factors, potentially masking or altering their relationship to the subjective experience of pain. Therefore, interpreting facial expressions in the context of pain requires careful consideration of these potential confounding factors.

## 4. Multimodal Pain Assessment Approaches

Pain assessment relies on a combination of physiological, behavioral, and self-report measures, with their weighting varying based on the clinical context. In acute pain, physiological and behavioral measures are more prominent due to the direct link between tissue damage and the pain experience [24,34]. Self-report remains valuable for assessing pain intensity and quality [45]. Conversely, chronic pain assessment emphasizes self-report measures to capture the biopsychosocial impact of pain, while physiological measures become less reliable [46]. For communicative patients, self-report is the gold standard However, in non-communicative patients, clinicians prioritize behavioral observations and physiological indicators, though the latter are not pain-specific [34]. Context-specific measures, such as observing specific behaviors, and multidimensional assessments are crucial for a comprehensive understanding of the pain experience [47].

By combining self-report measures with physiological markers and behavioral observations, a comprehensive understanding of the pain experience might be achieved. Therefore, a thorough pain assessment should consider these multiple factors [48]. This approach focuses on verbal and nonverbal communication, highlighting the importance of considering the entire patient interaction in pain assessment. This holistic approach should help to effectively evaluate and manage musculoskeletal pain.

Given the limitations of individual pain assessment methods, a multimodal approach combining multiple modalities, such as self-report, physiological measures, and behavioral observations, has been proposed as a more comprehensive way to assess musculoskeletal pain.

## 5. Neuroimaging Techniques

Advancements in neuroimaging technologies like fMRI (functional magnetic resonance imaging) and PET (positron emission tomography) have promoted the understanding of pain perception. fMRI, by detecting changes in blood flow in the brain, helps identify brain regions and neural pathways involved in pain processing [49]. It can objectively image the subjective effects of pain, including sensory, emotional, and motor components [50]. PET scans can be used to measure and quantify brain metabolic responses to pain [51,52].

While fMRI and PET provide valuable information about brain activity, caution is required when interpreting these images in the context of pain. This requires careful consideration of the complex interplay between nociception, pain perception, and individual variability [53].

An advanced application of neuroimaging is the development of pain-specific “brain signatures” or “pain biomarkers” using machine learning algorithms [54]. These objective markers could potentially differentiate between pain and non-pain states and even quantify pain intensity.

While fMRI shows promise in pain research and may offer objective insights into pain processing, it is not yet a perfect diagnostic tool with well-defined sensitivity and specificity values. Using fMRI as a pain detector raises concerns about privacy, autonomy, and potential coercion [54]. Individuals may feel pressured to undergo fMRI scans, and the results could be used against them in legal settings. fMRI is not yet a definitive measurement of pain, and research continues to explore its potential for objective pain assessment [53]. Combining fMRI with machine learning techniques may improve its accuracy and reliability [55]. Therefore, while fMRI holds promise as a tool for understanding pain, its use as a pain detector imposes ethical and legal challenges. Further research and careful consideration of these issues are necessary before fMRI can be reliably used in forensic contexts [56].

Overall, there is no data on specific sensitivity and specificity figures for combined physiological and behavioral measures in pain evaluation, as it highly depends on the specific combination of measures used, the population studied, and the criteria for defining pain. However, while combined physiological and behavioral approaches can improve pain assessments, more research is needed to determine the optimal combinations of measures and establish their sensitivity and specificity in different contexts.

## 6. Discussion

The objective measurement of musculoskeletal pain remains an active and crucial area of research. While significant progress has been made in physiological and neuroimaging-based approaches, pain’s multifaceted and subjective nature presents ongoing challenges (Table 1). Until more objective physiological and neurological measurement techniques are perfected, clinicians and researchers must rely on a combination of approaches, including self-report measures, which remain a cornerstone of pain assessment [13].

While physiological measures such as heart rate variability and skin conductance offer potential insights into pain-related autonomic responses, their interpretation can be confounded by various factors [12]. Similarly, although neuroimaging techniques like fMRI allow for the investigation of pain-related brain activity, translating these findings into clinically useful tools requires further validation and standardization [57].

The complexity of pain underscores the need for continued research to develop more robust, clinically applicable measurement tools. Integrating multiple modalities, including physiological, behavioral, and neuroimaging data, may provide a more comprehensive and reliable assessment of pain [58]. Developing objective measures holds immense potential for improving pain management strategies, particularly in populations who struggle to communicate their pain effectively, such as infants, nonverbal individuals, critically injured patients, and those under sedation who often cannot effectively communicate their symptoms [59].

The goal is to create objective pain assessment tools that are accurate and reliable but also user-friendly, cost-effective, and readily integrated into clinical practice, leading to more personalized and effective pain management.

While physiological and neuroimaging-based measures hold promise for objective pain assessment, several limitations and challenges remain. One key challenge is the influence of contextual factors on physiological responses. Factors like medication, underlying health conditions, and psychological states (such as stress or fear) can influence physiological measures like heart rate variability and skin conductance, making it difficult to isolate pain-specific responses. This highlights the importance of considering the individual’s context when interpreting physiological data [13].

Individual variability in pain perception and processing represents another major challenge because of the subjective nature of pain and the complex interplay between nociception, pain perception, and individual differences in brain structure and function [53]. This variability makes it difficult to establish universal pain biomarkers or “brain signatures” that apply to all individuals. Even with advanced neuroimaging techniques, there are limitations in interpreting brain activity in relation to pain. Acknowledging the challenges in establishing universal pain biomarkers due to individual variability [53], it is crucial to refer to the biopsychosocial model of pain explicitly. This model emphasizes that pain is not solely a biological phenomenon but arises from a complex interaction of biological, psychological, and social factors [60]. Individual differences in genetics, environment, psychological state, and cognitive variables all contribute to the subjective experience of pain [61]. Because the biopsychosocial perspective focuses attention on individual differences in the overall pain experience [62], and this intricate interplay dictates the variability in pain perception and processing, it becomes difficult to pinpoint a single “brain signature” applicable across all individuals [63]. Neuroimaging models must consider these socioeconomically situated and clinically relevant factors to increase their diagnostic relevance [64].

When neuroimaging-based pain signatures are considered, they need further validation and standardization across different populations and pain conditions before they can be reliably used in clinical practice. The current reliance on self-report measures, while imperfect, remains essential due to the inherent complexity of pain assessment [13].

## 7. Potential Future Directions

Despite these challenges, objective pain assessment methods hold great potential. In a translational research setting, physiological and neuroimaging techniques can deepen our understanding of pain mechanisms and help identify potential therapeutic targets by coordinating research efforts among basic scientists, clinical investigators, and pain medicine practitioners [65].

This goal might be achieved by exploring the potential of neuroimaging-based biomarkers for predicting treatment response, identifying individuals at risk of developing chronic pain, and personalizing pain management strategies [58].

In clinical practice, objective measures could complement self-report measures and improve diagnostic accuracy, particularly in patients with difficulty communicating pain, such as young children or individuals with cognitive impairments [65]. Further research and development are needed to overcome the current limitations and translate these promising techniques into practical clinical tools that can improve the lives of individuals suffering from pain. The integration of multiple modalities, like physiological, behavioral, and neuroimaging data, offers a promising approach to achieving more comprehensive and reliable pain assessments [58].

Pain can significantly interfere with a patient’s ability to participate in and progress through rehabilitation programs. By integrating objective measures, clinicians can gain a more comprehensive understanding of a patient’s pain experience and its impact on their functional abilities. Objective measures can help differentiate between actual functional limitations and those arising from pain-related avoidance or fear [34]. This distinction is crucial for accurately assessing a patient’s progress and tailoring interventions accordingly. By providing quantifiable data on pain levels and their physiological correlations, objective measures can inform decisions regarding the management of medication, prescribing exercise, and other therapeutic modalities. Objective data can facilitate communication between patients, therapists, and physicians, leading to more collaborative and patient-centered care. Seeing improvements in objective pain measures can motivate patients to adhere to their rehabilitation programs and actively participate in their recovery. While the subjective nature of pain poses a challenge to purely objective measurement, integrating these measures alongside traditional self-report and behavioral assessments can lead to more effective and personalized rehabilitation strategies.

Therefore, since objective pain measurement remains a complex challenge, future research should focus on integrating multiple modalities, including physiological, behavioral, and neuroimaging data, as well as incorporating patient-reported outcomes. This integrated approach and personalized considerations are crucial for developing clinically relevant and practical pain assessment tools [58].

Building on the integration of multiple modalities for objective pain measurement, future research should adopt a broader framework of precision medicine. Precision medicine aims to tailor treatment and prevention strategies to individual characteristics [61]. In the context of pain, this involves considering individual genetic, environmental, and lifestyle factors.

Digital biomarkers, derived from wearable sensors, mobile devices, and other digital technologies, offer the potential for the continuous, real-world monitoring of pain-related parameters [66]. When combined with big data analytics and machine learning, these digital biomarkers can help identify patterns and predict individual responses to treatment [55]. A digital twin, which creates a patient-specific representation of the musculoskeletal system by integrating heterogeneous data sources, is also a valuable option [67]

By integrating physiological, behavioral, neuroimaging, and patient-reported data within a precision medicine framework, more clinically relevant and practical pain assessment tools can be potentially created [58]. This personalized approach, which leverages the power of digital biomarkers and big data analytics, holds the key to enhancing pain management and improving patient outcomes.

## 8. Conclusions

The objective measurement of pain in musculoskeletal conditions remains a significant challenge but holds immense promise for improving diagnoses and treatments. While substantial advancements have been made in physiological and neuroimaging techniques, the subjective and multifactorial nature of pain continues to pose obstacles. Current objective measures show potential but are not readily available or sufficiently validated to replace the crucial role of patient self-reporting. Integrating multiple assessment modalities, alongside personalized considerations for individual patient experiences, represents the most promising approach for developing more effective and clinically relevant pain assessment tools.

## Figures and Tables

**Table 1:** Summary of objective assessment methods for musculoskeletal pain.

Assessment Modality	Method(s) Used	Mechanism	Advantages	Limitations	Clinical Utility	References
Physiological	HR, BP, HRV, Skin conductance	Autonomic response to pain stimuli	Non-invasive, continuous, accessible	Non-specific, affected by confounders	Adjunct for monitoring under sedation	[14,15]
Physiological	Pupillometry	Pain induces pupillary dilation	Objective, quick response	Lighting and medications can affect accuracy	Analgesia titration	[16,17]
Physiological	Biochemical markers (cortisol, cytokines)	Pain alters stress and immune biomarkers	Reflects systemic pain-related responses	Low accuracy, time lag	Biomarkers for research of chronic pain	[18,19]
Physiological	Nociceptive flexion reflex (EMG)	Spinal reflex triggered by nociceptive input	Quantifies spinal nociceptive processing	Requires setup, variable tolerability	Research, depth of analgesia studies	[20]
Physiological	Quantitative sensory testing	Pain alters sensory thresholds	Quantifiable, maps altered sensation	Time-consuming, user-dependent	Pain mechanism evaluation, tracking	[21,22]
Behavioral	Facial expression coding (FACS), AI analysis	Pain induces characteristic facial changes	Nonverbal, automation is possible	Cultural variation requires training	Infants, cognitive impairment	[25,26]
Behavioral	Body movements, posture, gait	Pain alters movement to avoid discomfort	Functional	Not pain-specific, hard to standardize	Rehab tracking, postural assessment	[27,28]
Behavioral	Functional performance tests	Pain impairs functional capacity	Standardized, widely used	Influenced by fitness, motivation	Outcomes in chronic pain and rehabilitation	[30]
Behavioral	Observer-based pain scales (e.g., FLACC)	Observable behavior reflects pain	Standardized and validated for nonverbal patients	Scoring is semi-subjective	Pain in nonverbal or pediatric settings	[34,35]
Neuroimaging	fMRI	Pain activates specific brain regions	High spatial resolution, brain-level analysis	Expensive, motion-sensitive	Pain diagnosis research, biomarker identification	[36,37]
Neuroimaging	PET	Pain alters metabolic/receptor activity	Molecular insights, receptor-specific imaging	Radiation exposure, experimental tracers	Pharmacologic pain research	[38]
Neuroimaging	EEG, pain-evoked potentials	Pain induces cortical EEG changes	Portable, fast, good temporal resolution	Low spatial resolution	Depth of analgesia	[36,37]
Neuroimaging	fNIRS	Pain alters cortical blood oxygenation	Portable, bedside-ready	Limited depth, validation needed	Pain tracking in rehab, infants	[19]
Computational	AI-based multimodal systems	AI detects multimodal patterns of pain	Automated, integrates multiple modalities	Not yet clinically validated	Potential future tool for continuous pain monitoring	[23,24]

HR—heart rate, BP—blood pressure, HRV—heart rate variability EMG—electromyography, AI—artificial intelligence, FLACC—the Face, Legs, Activity, Cry, and Consolability, EEG—electroencephalography, fMRI—functional magnetic resonance imaging, PET—positron emission tomography, Fnirs—Functional Near-Infrared Spectroscopy.

## Data Availability

Not applicable.

## References

[1] Goldsmith E.S., Murdoch M., Taylor B., Greer N., MacDonald R., McKenzie L., Rosebush C. (2017). Rapid Evidence Review: Measures for Patients with Chronic Musculoskeletal Pain. https://www.ncbi.nlm.nih.gov/books/NBK525003/.

[2] MacKichan F., Wylde V., Dieppe P. (2008). The assessment of musculoskeletal pain in the clinical setting. Rheum. Dis. Clin. N. Am..

[3] Chu Y., Zhao X., Han J., Su Y. (2017). Physiological Signal-Based Method for Measurement of Pain Intensity. Front. Media.

[4] Younger J., McCue R., Mackey S. (2009). Pain outcomes: A brief review of instruments and techniques. Curr. Pain Headache Rep..

[5] Salaffi F., Ciapetti A., Carotti M. (2012). Pain assessment strategies in patients with musculoskeletal conditions. Reumatismo.

[6] Misailidou V., Malliou P., Beneka A., Karagiannidis A., Godolias G. (2010). Assessment of patients with neck pain: A review of definitions, selection criteria, and measurement tools. J. Chiropr. Med..

[7] Kroenke K., Krebs E.E., Turk D., Von Korff M., Bair M.J., Allen K.D., Sandbrink F., Cheville A.L., DeBar L., Lorenz K.A. (2019). Core Outcome Measures for Chronic Musculoskeletal Pain Research: Recommendations from a Veterans Health Administration Work Group. Pain Med..

[8] Litcher-Kelly L., Martino S.A., Broderick J.E., Stone A.A. (2007). A systematic review of measures used to assess chronic musculoskeletal pain in clinical and randomized controlled clinical trials. J. Pain.

[9] Ong K.S., Seymour R.A. (2004). Pain measurement in humans. Surgeon.

[10] Campbell J.A., Lahuerta J. (1983). Physical Methods used in Pain Measurements: A Review. J. R. Soc. Med..

[11] Noble B., Clark D., Meldrum M., ten Have H., Seymour J., Winslow M., Paz S. (2005). The measurement of pain, 1945-2000. J. Pain Symptom Manag..

[12] Cowen R., Stasiowska M.K., Laycock H., Bantel C. (2015). Assessing pain objectively: The use of physiological markers. Anaesthesia.

[13] Subramaniam S.D., Doss B., Chanderasekar L.D., Madhavan A., Rosary A.M. (2018). Scope of physiological and behavioural pain assessment techniques in children—A review. Healthc. Technol. Lett..

[14] Sokolskiy V.M., Petrova I.Y., Sokolskiy M.V. (2019). Automated system for measuring integral pain index of patients during general anesthesia. 2019 International Scientific Conference on Digital Economy.

[15] Kanao-Kanda M., Kanda H., Iida T., Kikuchi S., Azuma N. (2021). Clinical Application of Laser Speckle Flowgraphy to Assess Changes in Blood Flow to the Foot After a Lumbar Sympathetic Ganglion Block: A Case Report. J. Pain Res..

[16] Morf S., Amann-Vesti B., Forster A., Franzeck U.K., Koppensteiner R., Uebelhart D., Sprott H. (2004). Microcirculation abnormalities in patients with fibromyalgia—Measured by capillary microscopy and laser fluxmetry. Arthritis Res. Ther..

[17] Chung K., Kim K.H., Kim E.D. (2018). Perfusion index as a reliable parameter of vasomotor disturbance in complex regional pain syndrome. Br. J. Anaesth..

[18] Loggia M.L., Juneau M., Bushnell C.M. (2011). Autonomic responses to heat pain: Heart rate, skin conductance, and their relation to verbal ratings and stimulus intensity. Pain.

[19] Kyle B.N., McNeil D.W. (2014). Autonomic arousal and experimentally induced pain: A critical review of the literature. Pain Res. Manag..

[20] Karpe J., Misiołek A., Daszkiewicz A., Misiołek H. (2013). Anaesthesiology And Intensive Care. A new method for the objective evaluation of postoperative pain. Kardiochirurgia i Torakochirurgia Polska/Pol. J. Thorac. Cardiovasc. Surgery.

[21] Storm H. (2008). Changes in skin conductance as a tool to monitor nociceptive stimulation and pain. Curr. Opin. Anaesthesiol..

[22] Linton S.J., Shaw W.S. (2011). Impact of psychological factors in the experience of pain. Phys. Ther..

[23] Brochard L., Martin G.S., Blanch L., Pelosi P., Belda F.J., Jubran A., Gattinoni L., Mancebo J., Ranieri V.M., Richard J.C. (2012). Clinical review: Respiratory monitoring in the ICU—A consensus of 16. Crit. Care.

[24] Fernandez Rojas R., Hirachan N., Brown N., Waddington G., Murtagh L., Seymour B., Goecke R. (2023). Multimodal physiological sensing for the assessment of acute pain. Front. Pain. Res..

[25] Skljarevski V., Ramadan N.M. (2002). The nociceptive flexion reflex in humans—Review article. Pain.

[26] Pasley J.D., O’Connor P.J. (2005). The nociception flexion (R-III) reflex: A potentially useful tool in exercise and pain studies. Int. J. Sport Exerc. Psychol..

[27] Kim H.Y., Gwak Y.S., Shim I. (2009). An electrophysiological method for quantifying neuropathic pain behaviors in rats: Measurement of hindlimb withdrawal EMG magnitude. J. Physiol. Sci..

[28] Lazaro R.P. (2015). Electromyography in musculoskeletal pain: A reappraisal and practical considerations. Surg. Neurol. Int..

[29] Haig A.J., Tong H.C., Yamakawa K.S.J., Quint D.J., Hoff J.T., Chiodo A., Miner J.A., Choksi V.R., Geisser M.E. (2005). The Sensitivity and Specificity of Electrodiagnostic Testing for the Clinical Syndrome of Lumbar Spinal Stenosis. Spine.

[30] Yalinay Dikmen P., Ilgaz Aydinlar E., Karlikaya G. (2013). Expected and Experienced Pain Levels in Electromyography. Noro Psikiyatr. Ars..

[31] Dworkin R.H., Turk D.C., Farrar J.T., Haythornthwaite J.A., Jensen M.P., Katz N.P., Kerns R.D., Stucki G., Allen R.R., Bellamy N. (2005). IMMPACT Core outcome measures for chronic pain clinical trials: IMMPACT recommendations. Pain.

[32] Smith S.M., Dworkin R.H., Turk D.C., McDermott M.P., Eccleston C., Farrar J.T., Rowbotham M.C., Bhagwagar Z., Burke L.B., Cowan P. (2020). Interpretation of chronic pain clinical trial outcomes: IMMPACT recommended considerations. Pain.

[33] Tranquilli C., Bernetti A., Picerno P. (2013). Ambulatory joint mobility and muscle strength assessment during rehabilitation using a single wearable inertial sensor. Med. Dello Sport.

[34] Prkachin K.M., Schultz I., Berkowitz J., Hughes E., Hunt D. (2002). Assessing pain behaviour of low-back pain patients in real time: Concurrent validity and examiner sensitivity. Behav. Res. Ther..

[35] Ekman P., Friesen W.V. (1976). Measuring facial movement. J. Nonverbal Behav..

[36] Lints-Martindale A.C., Hadjistavropoulos T., Barber B., Gibson S.J. (2007). A Psychophysical Investigation of the Facial Action Coding System as an Index of Pain Variability among Older Adults with and without Alzheimer’s Disease. Pain Med..

[37] Lautenbacher S., Walz A.L., Kunz M. (2018). Using observational facial descriptors to infer pain in persons with and without dementia. BMC Geriatr..

[38] Wolf K. (2015). Measuring facial expression of emotion. Dialogues Clin. Neurosci..

[39] Egede J., Song S., Olugbade T., Wang C., Williams A.C.d.C., Meng H., Aung M.H., Lane N.D., Valstar M., Bianchi-Berthouze N. EMOPAIN Challenge 2020: Multimodal Pain Evaluation from Facial and Bodily Expressions. Proceedings of the 2020 15th IEEE International Conference on Automatic Face and Gesture Recognition (FG 2020).

[40] Gjoreski M., Kiprijanovska I., Stankoski S., Mavridou I., Broulidakis M.J., Gjoreski H., Nduka C. (2022). Facial EMG sensing for monitoring affect using a wearable device. Sci. Rep..

[41] Lian Z., Guo Y., Cao X., Li W. (2021). An Ear Wearable Device System for Facial Emotion Recognition Disorders. Front. Bioeng. Biotechnol..

[42] Kwon J., Ha J., Kim D.-H., Choi J.W., Kim L. (2021). Emotion Recognition Using a Glasses-Type Wearable Device via Multi-Channel Facial Responses. IEEE Access.

[43] Nandini D., Yadav J., Singh V., Mohan V., Agarwal S. (2025). An ensemble deep learning framework for emotion recognition through wearable devices multi-modal physiological signals. Sci. Rep..

[44] Kallipolitis A., Galliakis M., Menychtas A., Maglogiannis I. (2020). Affective analysis of patients in homecare video-assisted telemedicine using computational intelligence. Neural Comput. Appl..

[45] Robinson C.L., Phung A., Dominguez M., Remotti E., Ricciardelli R., Momah D.U., Wahab S., Kim R.S., Norman M., Zhang E. (2024). Pain Scales: What Are They and What Do They Mean. Curr. Pain Headache Rep..

[46] Jensen B. (2016). Chronic pain assessment from bench to bedside: Lessons along the translation continuum. Transl. Behav. Med..

[47] Hassett A.L., Whibley D., Kratz A., Williams D.A. (2020). Measures for the Assessment of Pain in Adults. Arthritis Care Res..

[48] Roberts L., Bucksey S.J. (2007). Communicating with patients: What happens in practice?. Phys. Ther..

[49] Becerra L., Borsook D. (2006). Insights into pain mechanisms through functional MRI. Drug Discov. Today Dis. Mech..

[50] Fomberstein K., Qadri S., Ramani R. (2013). Functional MRI and pain. Curr. Opin. Anaesthesiol..

[51] Mehta V., Bouchareb Y., Ramaswamy S., Ahmad A., Wodehouse T., Haroon A. (2020). Metabolic Imaging of Pain Matrix Using 18F Fluoro-deoxyglucose Positron Emission Tomography/Computed Tomography for Patients Undergoing L2 Dorsal Root Ganglion Stimulation for Low Back Pain. Neuromodulation.

[52] Boissoneault J., Sevel L., Letzen J., Robinson M., Staud R. (2017). Biomarkers for Musculoskeletal Pain Conditions: Use of Brain Imaging and Machine Learning. Curr. Rheumatol. Rep..

[53] Davis K.D. (2016). Legal and ethical issues of using brain imaging to diagnose pain. Pain Rep..

[54] Opancina V., Sebek V., Janjic V. (2024). Advanced neuroimaging and criminal interrogation in lie detection. Open Med..

[55] van der Miesen M.M., Lindquist M.A., Wager T.D. (2019). Neuroimaging-based biomarkers for pain: State of the field and current directions. Pain Rep..

[56] Farah M.J., Hutchinson J.B., Phelps E.A., Wagner A.D. (2014). Functional MRI-based lie detection: Scientific and societal challenges. Nat. Rev. Neurosci..

[57] Berger S.E., Baria A.T. (2022). Assessing Pain Research: A Narrative Review of Emerging Pain Methods, Their Technosocial Implications, and Opportunities for Multidisciplinary Approaches. Front. Pain. Res..

[58] Mackey S., Greely H.T., Martucci K.T. (2019). Neuroimaging-based pain biomarkers: Definitions, clinical and research applications, and evaluation frameworks to achieve personalized pain medicine. Pain Rep..

[59] Guaragni B., Howell A., Rehman F.U., Jain A. (2013). Management of pain in ventilated neonates: Current evidence. Paediatr. Child Health.

[60] Gatchel R.J. (2015). The Continuing and Growing Epidemic of Chronic Low Back Pain. Healthcare.

[61] Daneshjou R., Brenner S.E., Chen J.H., Crawford D.C., Finlayson S.G., Kidziński Ł., Bulyk M.L. (2022). Precision Medicine: Using Artificial Intelligence to Improve Diagnostics and Healthcare. Pac. Symp. Biocomput..

[62] Gatchel R.J., Peng Y.B., Peters M.L., Fuchs P.N., Turk D.C. (2007). The biopsychosocial approach to chronic pain: Scientific advances and future directions. Psychol. Bull..

[63] Kohoutová L., Atlas L.Y., Büchel C., Buhle J.T., Geuter S., Jepma M., Koban L., Krishnan A., Lee D.H., Lee S. (2022). Individual variability in brain representations of pain. Nat. Neurosci..

[64] Reddan M.C. (2021). Recommendations for the Development of Socioeconomically-Situated and Clinically-Relevant Neuroimaging Models of Pain. Front. Neurol..

[65] Mao J. (2009). Translational pain research: Achievements and challenges. J. Pain.

[66] Rani B., Gupta M., Ganesh V., Sharma R., Bhatia A., Ghai B. (2025). Efficacy of mobile health interventions in the conservative management of chronic low back pain in low- and middle-income countries: A systematic review, meta-analysis, and trial sequential analysis. Pain Rep..

[67] De Domenico M., Allegri L., Caldarelli G., d’Andrea V., Di Camillo B., Rocha L.M., Rozum J., Sbarbati R., Zambelli F. (2025). Challenges and opportunities for digital twins in precision medicine from a complex systems perspective. NPJ Digit. Med..

